# Two of us live in this body

**DOI:** 10.1192/j.eurpsy.2022.2256

**Published:** 2022-09-01

**Authors:** M. Palomo Monge, M. Pérez Fominaya, M.V. López Rodrigo, A. Osca Oliver, M.F. Tascón Guerra, C. García Montero, V. Ros Font

**Affiliations:** 1 Hospital Nuestra Señora del Prado, Psiquiatria, Talavera de la Reina, Spain; 2 Hospital Nuestra Señora del Prado, Psiquiatría, Talavera de la Reina, Spain; 3 Hospital Provincial de Ávila, Servicio De Psiquiatría, Ávila, Spain

**Keywords:** Dissociative Identity disorder, multiple personality, dissociative disorders, delusional symptoms

## Abstract

**Introduction:**

The dissociative disorders are characterized by a disruption of and/or discontinuity in the normal integration of consciousness, memory, identity, emotion, perception, body representation, motor control, and behavior.

**Objectives:**

We present the case of a 22-year-old patient, who has been following up for just over a year. The patient refers that two people inhabit her body, talk to each other, exchange opinions and both have control over the body, one giving the turn to the other depending on the circumstances. To this is added delusional symptoms of grandeur and sensorial-perceptual symptoms. In turn, depressive symptoms have appeared that have led the patient to have several suicide attempts throughout the follow-up time.

**Methods:**

During this time, the patient has required hospital admission on two occasions due to the autolytic ideation. Treatment with neuroleptics and antidepressants has been established that have helped control delusions and thoughts of death, but not the dissociative clinic.

**Results:**

Dissociative Identity disorder 300.14 (F44.81)

**Conclusions:**

The different symptoms presented by the patient, as well as the social and occupational deterioration that he presents, make this an extremely complicated case, both in diagnosis and in treatment. Dissociative identity disorder has been very controversial, changing its diagnostic criteria over time. More studies are needed and perhaps future research can give us more clues about this disorder.

**Disclosure:**

No significant relationships.

